# Rare Germline Variants in the Adenomatous Polyposis Coli Gene Associated with Dental and Osseous Anomalies

**DOI:** 10.3390/ijms25158189

**Published:** 2024-07-26

**Authors:** Gergely Büki, Gréta Antal, Judit Bene

**Affiliations:** 1 Department of Medical Genetics, Clinical Center, Medical School, University of Pécs, 7624 Pécs, Hungary; buki.gergely@pte.hu; 2 Department of Dentistry, Oral and Maxillofacial Surgery, Clinical Center, Medical School, University of Pécs, 7623 Pécs, Hungary; antal.greta@pte.hu

**Keywords:** *APC* gene, osteoma, dental abnormalities, supernumerary teeth, Wnt signaling

## Abstract

*APC* is a tumor suppressor gene that exerts its effect through the regulation of the Wnt signaling pathway. Loss of function mutations of the gene are associated with familial adenomatous polyposis (FAP). Early diagnosis in FAP patients is essential to prevent the development of colorectal cancer. Extraintestinal manifestations often precede the formation of the polyposis; therefore, these manifestations may serve as a clinical indicator for the condition. The aim of this study was to assess genotype–phenotype associations between the location of *APC* mutations and various extraintestinal features, mainly focusing on osseous and dental anomalies. Analyses of our cases and the mutations available in the literature with these manifestations revealed that mutations in the N-terminal region (amino acids 1–~1000) of the protein are more frequently associated with only osseous anomalies, whereas dental manifestations are more prevalent in mutations in the middle region (amino acids 1000–~2100). In addition, supernumerary teeth were found to be the most common dental feature. Since dental abnormalities often precede intestinal polyposis, dentists have a crucial role in the early identification of patients at risk.

## 1. Introduction

The human adenomatous polyposis coli (APC) gene located on chromosome 5q21-q22 encodes a 300 kDa, ubiquitously expressed tumor suppressor protein [1]. The gene consist of 15 exons and plays a crucial role in preserving the colonic epithelial structure [2]. In addition to its main function as an antagonist of the Wnt pathway, APC is essential, as it interacts with a number of different proteins. Through its complex function, the APC protein is involved in cell adhesion and migration, spindle assembly, neuronal differentiation, chromosome segregation and cell cycle control [1,3,4,5].

Since the identification and characterization of the gene [6], a lot of different research has been carried out. It has been revealed that APC is involved in the maturation process of the epithelial cells of the colon, and its expression increases from the base of crypt to the top of the villi, extending into the lumen [2,6]. Mutations in the *APC* gene can induce the development of numerous adenomatous polyps, therefore contributing to the development of colorectal cancers (CRC) [6]. According to Hankey et al. [7], biallelic mutations in the *APC* gene are responsible for 45–80% of CRC, which is the second leading cause of cancer deaths.

The loss of APC function has been associated with multiple disorders [8,9]. The most well-known among these is the classic familial adenomatous polyposis (FAP). The estimated prevalence for FAP is approximately 1 in 8000 to 1 in 18,000, with a worldwide distribution [10]. It is estimated that in about 25% of FAP patients, de novo mutations develop the disorder [10]. In addition, large deletions may account for approximately 15% of the cases [11]. It follows an autosomal dominant inheritance pattern and is characterized by hundreds to thousands of adenomatous polyps in the colon and rectum. They usually appear during childhood or adolescence [12,13]. According to previous studies, the development of adenomas requires inactivating mutations in both *APC* alleles, which typically result from an inherited and a somatic mutation [14]. The vast number of early-onset adenomas carry an increased risk of CRC. The number of polyps is generally related to the risk of developing cancer [15]. The mean age of the development of polyps is 16 years, and generally, patients are diagnosed between 20 and 40 years of age. In addition, extraintestinal manifestations may also occur in 70% of FAP patients [16], such as odontomas, osteomas, congenital hypertrophy of the retinal pigment epithelium (CHRPE), papillary carcinoma of the thyroid, hepatoblastoma, medulloblastoma, desmoid, soft tissue, gastrointestinal, and benign tumors, and various dental findings [17,18]. Although almost complete penetrance of colonic polyposis can be observed, the extracolonic manifestations display variable penetrance and severity [10].

If fewer than 100 adenomatous polyps appear, a phenotypic variant of FAP known as attenuated FAP (AFAP) can be differentiated [19]. Compared to the classic FAP, adenomas appear later and are more proximally distributed in the colon. Nevertheless, cancer is diagnosed at a later age and, predominantly, the risk of colorectal cancer is slightly lower [20]. Compared to FAP patients, generally fewer extraintestinal manifestations occur.

In addition to classic and attenuated FAP, other APC-associated polyposis conditions are also known, such as gastric adenocarcinoma and proximal polyposis of the stomach (GAPPS), Turcot syndrome (associates FAP with central nervous system tumors), and Gardner syndrome (GS, association of FAP with osteomas and soft tissue tumors). Moreover, *APC* mutations have been associated with various neurological disorders and intellectual disabilities, as well [21,22,23].

Gardner syndrome [24] is a variant of FAP that is mainly characterized by a triad of multiple gastrointestinal polyps, cutaneous and subcutaneous soft tissue tumors, and osteomas. The prevalence of GS is estimated to range from 1 in 7000 to 1 in 30,000 live births, with variable expressivity, affecting women and men equally [25]. Familial accumulation has been observed in most cases, but approximately 20–30% of cases occur de novo [25,26]. Osteomas are one of the most frequent bone alterations, which most commonly affect the maxilla, mandible, and/or the frontal bone [25,27,28]. In comparison, orbital osteomas are extremely rare [25]. The gastrointestinal symptoms, such as diarrhea, anemia, lower gastrointestinal bleeding, abdominal pain, etc., occur around the age of 30 [26]. More than 70% of patients will develop extracolonic features, such as neoplastic lesions, CHRPE, fibromas, thyroid cancer, lipomas, gastric fundic gland polyps, juvenile nasopharyngeal angiofibromas, dental abnormalities, epidermoid cysts, or desmoid, solid organ, and/or brain tumors [16,25,26]. Desmoid tumors, which may cause life-threatening complications, develop in approximately 3.5–5.7% of GS patients, usually at the retroperitoneum or the abdominal cavity [29]. CHRPE is present in about 90% of the patients [29]. Approximately 30–70% of GS patients present with dental abnormalities, such as odontomas, impacted/obstructed/unerupted teeth, osteomas of the jaw, missing or supernumerary teeth, or other abnormal tooth morphology [30,31]. The presence of both dental abnormalities and osteomas is suggestive of underlying GS [32,33,34,35].

Certain extraintestinal manifestations in GS, such as dento-osseous features often precede the intestinal polyposis; therefore, they can potentially serve as an early marker of this syndrome. Previous research has stated that bone and cutaneous abnormalities develop approximately 10 years before the onset of polyposis [36,37]. Altogether, the specific extraintestinal manifestations may help in the early diagnosis and proper treatment of GS [26].

According to the ClinVar database, approximately 12,500 mutations have been observed within the *APC* gene, of which roughly 2500 are pathogenic or likely pathogenic variants. The majority of the mutations are intragenic, small-scale variants (<50 base pairs) manifesting as nonsense, frameshifts, deletions, or insertions that could lead to a premature stop codon, thus potentially resulting in either a truncated protein or nonsense-mediated decay. A wide variety of mutations have been observed throughout the *APC* gene, but the majority (~75%) of both germline and somatic mutations occur in the 15th exon of the *APC* gene [38]. A mutational cluster region (MCR) containing about 60% of all identified mutation sites has been defined [39], which localizes between amino acids 1000 and 1600 [7]. In addition to MCR, other mutational hotspots can be distinguished, and several specific genotype–phenotype associations have been demonstrated between the location of a mutation and the presence, frequency, and/or severity of particular manifestations [40,41]. These investigations mainly focused on colonic manifestations. Regarding dental anomalies, only a few studies have discussed the genotype related to the dental phenotype [40,42].

In our study, we aimed to study the association between *APC* mutations and dental or osseous manifestations. For this purpose, we collected *APC* mutations accompanying dental and/or osseous manifestations published in the literature and added two of our cases. In order to find a link between the dental or osseous symptoms and the *APC* mutations, we have analyzed the localizations of various mutations, their possible role in the protein function, and the observed manifestations.

## 2. Results

Altogether, 49 different cases (with 30 various mutations), including our two cases, have been found with various dental and/or osseous abnormalities in which the presence of an *APC* mutation was approved (Table 1). Based on the location of the mutation (N-terminal or middle region of the protein), the individuals with dental and/or osseous anomalies were classified into two groups. Almost half of the patients (n = 23) carried a mutation in the N-terminal region; among these, 15 (~65%) cases presented only osseous abnormalities, 4 (~17%) individuals showed only dental abnormalities, and 4 (~17%) patients had both dental and osseous anomalies. In 24 patients, the mutation was located in the middle region, of which 7 (~29%) demonstrated only osseous abnormalities, 9 (~38%) cases displayed only dental anomalies, and 8 (~33%) patients had both dental and osseous anomalies. Two patients with mutations localized in the C-terminal region presented only dental anomalies; they were not involved in the statistical analyses.

In order to ascertain a potential influence of mutation localization on specific dental or osseous anomalies, a comparative analysis between patients with mutations in the N-terminal or middle region was conducted. The following manifestations were involved in osseous anomalies: osteomas (OS); dense bone island (DBI); and hazy sclerosis (HS). In the case of dental anomalies, mesiodens; supernumerary teeth (ST); impacted teeth (IT); odontomas (OD); and other dental abnormalities were included.

Mutations in the N-terminal region are more frequently associated with only osseous anomalies (15 out of 23) compared to mutations in the middle region (7 out of 24). A statistical analysis further confirmed this association with a significant difference (*p* = 0.014). Notably, osteoma was the predominant symptom in osseous anomalies and was observed across both regions in 20 out of 22 cases (~91%).

Dental manifestations appeared to be more prevalent among cases with mutations in the middle region (17 cases out of 24) compared to those in the N-terminal region (8 cases out of 23). A significant difference (*p* = 0.014) was observed in the frequencies. Furthermore, an examination of specific dental manifestations has been carried out. Mesiodens are a type of supernumerary teeth; therefore, in our calculations, they were treated together. A significant difference was observed in the frequencies of the supernumerary teeth (*p* = 0.030) and the impacted teeth (*p* = 0.042) presented in the patients with a mutation located in the middle region compared to the N-terminal.

Although twice as many cases (8 out of 24 vs. 4 out of 23) presented dental abnormalities with osseous anomalies, and nearly twice as many (7 out of 24 vs. 4 out of 23) demonstrated only dental anomalies, the statistical analysis could not determine a significant difference in either case (*p* = 0.180, *p* = 0.272, respectively).

Among dental cases, supernumerary teeth were present in 10 cases out of 17 (59%) in the middle domain and 3 out of 8 cases (37.5%) in the N-terminal domain; however, no significant difference was established (*p* = 0.278).

In conclusion, while mutations in the N-terminal domain appear to be predominantly associated with osseous anomalies, mutations in the middle domain may contribute to a higher incidence of dental anomalies. These findings underscore the potential role of mutation localization in determining the phenotypic expression of dental and osseous abnormalities.

Regarding the polyposis phenotype among the presented cases, the same number of patients were diagnosed with classic FAP and Gardner syndrome. The majority of patients with classic FAP carry mutations in the N-terminal region, whereas patients with Gardner syndrome harbor mutations localized in the middle region of the APC protein.

## 3. Discussion

The early diagnosis of individuals affected by FAP, a rare genetically determined disorder, is crucial to prevent the development of colorectal cancer. Moreover, presymptomatic diagnosis is essential, as two-thirds of symptomatic patients will already have developed carcinoma and consequently have a worse prognosis [58].

Since the disorder is inherited in an autosomal dominant manner, family members have a 50% risk of inheriting the disease. Therefore, genetic testing in individuals at risk has an important role in the identification of the disease-causing variants in the *APC* gene. Due to the state-of-the-art NGS-based technology, it is feasible nowadays; however, in the general population, it is not applicable because of ethical and other considerations. Furthermore, genetic analysis plays an important role in the early diagnosis of patients with atypical manifestations as was also the case in our patients. Our proband had initial atypical skeletal symptoms and dental anomalies only, and the diagnosis was established with the help of whole exome sequencing [57].

Extraintestinal manifestations, such as various dental and osseous abnormalities, accompanying FAP have a potential role as a clinical indicator in early diagnosis. It is well-known from previous studies [59] that these manifestations often precede the development of polyposis. Thakker et al. [60] developed a weighted dental panoramic radiograph score (DPRS) system as a diagnostic tool for individuals with high risk of FAP. DPRS took into consideration the nature, extent, and site of osseous and dental changes on dental panoramic radiographs in FAP patients and was shown to have a sensitivity of up to 69% and specificity of 100%. Using this tool, differences in the frequency of dental anomalies and osseous jaw lesions between FAP and unaffected groups were observed. Bone changes and dental anomalies were found in 81% and 37% of the FAP patients, respectively. Aggarwal and colleagues evaluated the validity of this DPRS tool on an independent patient group, and they found a 100% specificity. Within their patient cohort, osseous jaw lesions were seen in 69% of FAP patients, and dental anomalies were seen in 35% [58]. Bone alterations (osteomas, islands of bone condensation, and diffuse sclerosis) are more frequent than dental anomalies in FAP patients [43,47,58,60]. Almeida et al. performed a meta-analysis investigating the oral manifestations of FAP and their frequency in affected individuals. It was found that the frequency of osseous jaw lesions ranged from 21% to 95%, and the frequency of dental anomalies ranged from 9.3% to 56% in FAP patients [61]. The frequency of some dental and osseous anomalies was found to be higher in FAP patients than in the general population (Table 2).

In several studies, a genotype–phenotype association was performed to reveal an association between the location of *APC* mutations and extraintestinal manifestations in FAP patients. In their study, Septer and colleagues investigated pediatric patients with FAP [42]. They found that in patients carrying a mutation in or upstream of codon 1309, a higher frequency of osteomas (77.8%) and jawbone sclerosis (44.4%) was observed, and 77% of these patients had at least one dental anomaly. Moreover, they observed that osteomas were present in the jaw in 42.9% of genetic variants in the 5′-codon 516 region, 66.7% in variants between codons 849 and 1309, and 77.8% of genetic variants situated between codons 1310 and 3′. Previously, Bertario et al. found that mutations beyond codon 1444 were significantly associated with osteomas [66]. Another study by Bertario [67] revealed that dental abnormalities were strongly associated with osteomas, and both conditions were associated with mutations between codons 1256 and 1303. Davies et al. [40] observed an association of significantly more abnormalities on dental panoramic radiographs with mutations distal to codon 1444 compared to mutations at codons 1–1444. In a study by Bisgaard and colleagues, osteomas were only identified in patients with mutations between codons 767 and 1513 [68].

In this study, a mutation found in our patients was analyzed along with 29 previously published *APC* mutations in 47 patients. Our patients, who were carrying a c.4700C>G (p.Ser1567*) mutation presented with impacted teeth, odontomas, and osteomas. The detailed clinical description was published previously [57]. This mutation has been reported previously [69], but no dento-osseous anomalies were associated with it. A detailed clinical evaluation of dental and osseous anomalies of patients with known *APC* mutations revealed that osseous manifestations were more frequent than dental abnormalities (34 out of 46 vs. 24 out of 46, respectively), similar to previous findings [43,47,58,60]. Osteoma was the most frequent bone lesion, and supernumerary teeth were more prevalent among the dental anomalies. The investigation of genotype–phenotype associations in *APC* mutations and these extraintestinal manifestations revealed that mutations localized in the N-terminal region (1–1000 aa’s) of the APC protein are more frequently associated with only osseous anomalies compared to mutations localized in the middle region (1000–2100 aa’s) (65% vs. 32%). Moreover, dental manifestations were more frequently associated with mutations in the middle region than those in the N-terminal region (68% vs. 35%). Supernumerary teeth were found to be the most prevalent dental anomaly, and they were also found to be associated more frequently with mutations in the middle region compared to those in the N-terminal region.

**Figure 1:**
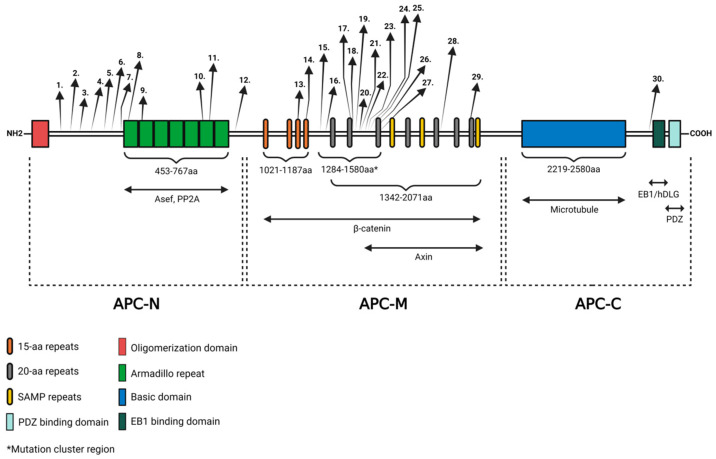
The structure of the APC protein and the locations of known *APC* mutations. APC contains regions required for oligomerization, armadillo repeats, β-catenin-binding domain, AXIN-binding domain, basic domain, EB1-binding domain, and HDLG-binding domain. Three major region names and the interacting partners of the protein are shown underneath the APC diagram. APC-N is the N-terminal region, APC-M is the middle region, and APC-C is the C-terminal region. Locations of the mutations are indicated by arrows. Numbers are in correspondence with the mutations listed in Table 1. The figure was created with BioRender.com (accessed on 29 March 2024).

APC is a multifunctional protein containing multiple functional regions, including specific repeats, definite motifs, nuclear localization, and export signals (Figure 1). The protein can be divided into three major sections known as the N-terminal (APC-N), middle (APC-M), and C-terminal (APC-C) regions. The N-terminal region contains amino acids 1–~1000, the middle region covers amino acids ~1000–2100 and the C-terminal region spans over amino acids 2130–2843 [70]. The structure of the protein provides binding sites for one or more different partner proteins, such as β-catenin, Axin, microtubule, human DISCS large protein (hDLG), and APC-stimulated guanine nucleotide exchange factor (Asef).

The N-terminal region contains an oligomerization domain and seven repeats, known as the armadillo region, which has been shown to bind to PP2A and Asef [17]. The middle region encompasses various repeats, such as 15- and 20-amino acid signature repeats and characteristic SAMP amino acid sequences. They provide binding sites for the Wnt signaling complex (β-catenin, Axin, and KIF3a). The C-terminal region contains a basic domain, which provides a microtubule-binding site, a PDZ-binding motif, and domains for the interaction between end-binding protein 1 (EB1) and hDLG. The protein interacts with multiple cytoskeletal proteins, thereby influencing various cellular processes, with special respect to the Wnt signaling pathway [1,17].

The role of Wnt in odontogenesis and bone development has been addressed previously [71]. Previous experiments on mouse models established that balanced Wnt/β-catenin activity is essential in tooth development. Disruption of the Wnt pathway leads to either impaired tooth formation or supernumerary teeth [72,73]. This was further demonstrated in research by Panyarat et al. [50], in which they hypothesized that as a result of mutations in the *APC* gene, the β-catenin levels will increase. They suggested that the reduction in CTBP interaction and disruption of axin or microtubule interaction will affect the WNT/β-catenin signaling.

**Figure 2:**
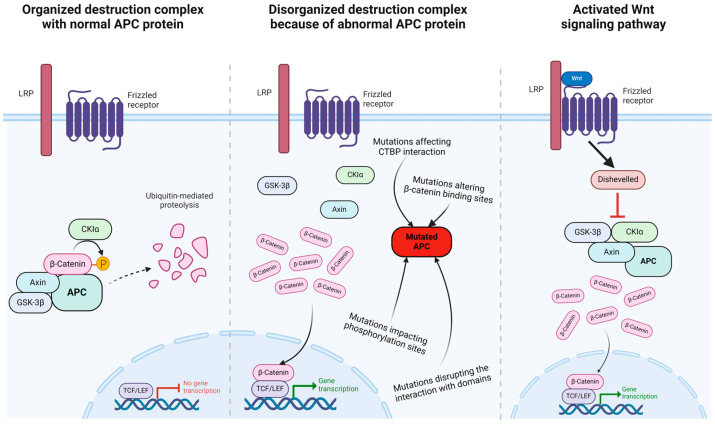
Mutations in APC protein mimic the effects of Wnt signaling pathway. The normal APC protein inactivates the transcriptional role of β-catenin through phosphorylation leading to ubiquitinylation and degradation (**left**), whereas mutated APC protein allows β-catenin to enter the nucleus (**middle**), similar to the result of an active Wnt signal transduction pathway (**right**). The figure was adapted from “Wnt Signaling Pathway Activation and Inhibition”, by BioRender.com (accessed on 29 March 2024). Retrieved from https://app.biorender.com/biorender-templates.

Normally β-catenin would be phosphorylated and broken down by a destruction complex [74]. The so-called β-catenin destruction complex consists of axin, β-catenin, and APC and phosphorylates β-catenin with the help of CK1α and GSK-3β [75]. Previous studies [17,74,76,77] have stated that the accumulation of β-catenin in the cellular cytoplasm and nucleus can occur in various cases, including the direct mutation of β-catenin as a result of the Wnt signal or the inactivation of APC. Truncation of the APC is assumed to disrupt the interaction of the destruction complex, thereby disrupting the degradation of β-catenin [75]. As a result of elevated β-catenin, it will form a complex with TCF/LEF in the nucleus and activate the gene expression of target genes [17] (Figure 2).

Our statistical analysis proved that patients with dental abnormalities were more pronounced in cases with a mutation in the middle region. Moreover, supernumerary teeth were more frequently associated with mutations in the middle region. The majority of the mutations in this region are frameshift or nonsense mutations resulting in the termination of protein translation that could lead to a non-functional APC protein or the nonsense-mediated decay of the transcript [47]. There are a few missense mutations that may cause the modification of the APC protein structure, which probably has an effect on its binding properties [50]. Numerous binding sites for the axin and β-catenin interaction are present in the middle region of the APC protein. Therefore, the mutations, especially in the middle domain, are expected to result in a defective APC protein formation, which can affect the assembly of the protein complex, thus contributing to an increased β-catenin level. The elevated β-catenin leads to increased gene expression, which may contribute to supernumerary tooth formation [1,17,72,73].

The limitations of our study are that we found only a limited number of genetic mutations in the *APC* gene with clear clinical information. Although several cases of Gardner syndrome have been published in the literature, their exact genetic variants are not available.

## 4. Materials and Methods

### 4.1. Literature Search and Keyword Specification

A relevant literature search was applied in the PubMed database using the following keywords and phrases: “dental anomalies” or “dental abnormalities” or “teeth abnormality” or teeth anomalies” or “osseous abnormalities” or “osseous anomalies” and “Gardner’s syndrome” or “*APC* gene mutation” or “familial adenomatous polyposis”. After a thorough analysis of potential publications with genotype–phenotype data, patients with dental and/or osseous abnormalities were collected from the literature. Dental anomalies included supernumerary teeth, ST; impacted teeth, IT; odontomas, OD; mesiodens; and other dental abnormalities. Osseous anomalies included osteomas, OS; dense bone island, DBI; and hazy sclerosis, HS.

### 4.2. Statistical Analysis

A statistical analysis was carried out to see the relationship between the location of mutations and specific manifestations of dental and/or osseous anomalies. A descriptive statistical analysis, including a chi-square test, was applied using SPSS version 27.0 (IBM Corp. Released 2020. IBM SPSS Statistics for Windows, Version 27.0. Armonk, NY, USA: IBM Corp.). The expected values for each cell were five or higher, and *p* < 0.05 was considered the level of significance.

## 5. Conclusions

Germline mutations of the *APC* gene are responsible for the development of FAP, a tumor predisposition syndrome. The early diagnosis of FAP is essential to prevent the development of colorectal cancer and improve the prognosis of the disease. Extraintestinal manifestations of FAP, such as osseous or dental anomalies, often precede the development of polyposis and may serve as a clinical marker for the presence of this condition. Dentists should be familiar with the typical dento-osseous features of this disorder and refer the patients to genetic counseling. In our study, we demonstrated that mutations in the N-terminal region of the protein are associated with osseous lesions more frequently, whereas mutations in the middle region are linked to dental anomalies. However, further studies are needed to better characterize the mutational spectrum of this gene and the associated extraintestinal features.

## Figures and Tables

**Table 1:** *APC* germline mutations with dento-osseous manifestations and FAP phenotype.

	Mutation		Gender/Age	Polyposis Phenotype	Colorectal Cancer	Extraintestinal Manifestations		Ref.	Localization
	DNA	Protein				Dental Anomalies	Osseous Anomalies		
1	c.481C>T ^a^	p.Gln161*	M/54	Classic FAP	+	-	Hs	[43]	APC-N-terminal region
	c.481C>T ^a^	p.Gln161*	F/20	Classic FAP	-	-	Os, Hs		
	c.481C>T ^a^	p.Gln161*	M/18	Classic FAP	-	IT, Od	Dbi		
2	c.532-1G>A ^b^	-	M/33	Classic FAP	+	-	Os, Hs		
	c.532-1G>A ^b^	-	F/28	Classic FAP	+	Od	Os		
	c.532-1G>A ^b^	-	M/7	Classic FAP	-	Od	Os, Hs, Dbi		
3	c.646C>T	p.Arg216*	F/12	ND	ND	IT, Od	-	[44]	
4	c.761C>G	p.Ser254*	-/-	Attenuated FAP	ND	ST	-	[45]	
5	c.839C>G	p.Ser280*	-/39	ND	ND	-	Os	[46]	
6	c.1240C>T	p.Arg414Cys	-/24	ND	ND	-	Os		
7	c.1354_1355delGT	p.Val452SerfsX7	-/-	Classic FAP	ND	-	Os	[45]	
8	c.1370C>G	p.Ser457*	M/39	Classic FAP	+	-	Os, Dbi	[47]	
	c.1370C>G ^c^	p.Ser457*	M/32	Classic FAP	-	-	Os, Hs, Dbi		
	c.1370C>G ^c^	p.Ser457*	F/34	Classic FAP	+	-	Hs, Dbi		
	c.1370C>G ^c^	p.Ser457*	M/11	Classic FAP	-	ST	Hs		
9	c.1495C>T	p.Arg499*	F/27	ND	-	-	Os	[48]	
10	c.2092T>G	p.Leu698*	M/23	Classic FAP	+	Dental abnormalities	-	[49]	
	c.2092T>G ^d^	p.Leu698*	M/48	Classic FAP	+	-	Os		
	c.2092T>G ^d^	p.Leu698*	M/23	Classic FAP	-	-	Os		
	c.2092T>G ^d^	p.Leu698*	F/24	Classic FAP	-	-	Os		
	c.2092T>G ^d^	p.Leu698*	M/21	Classic FAP	-	-	Os		
11	c.2138C>G	p.Ser713*	-/37	ND	ND	-	Os	[46]	
12	c. 2740T>G	p.Cys914Gly	M/-	ND	ND	Mesiodens	-	[50]	
13	c.3199_3202delCAAT	p.Ser1068Glyfs*57	-/-	Classic FAP	ND	-	Os	[45]	APC-Middle region
14	c.3374T>C	p.Val1125Ala	F/-	ND	ND	ST	-	[50]	
	c.3374T>C	p.Val1125Ala	M/-	ND	ND	mesiodens	-	[50]	
15	c.3880_3881delCA	p.Gln1294Glyfs*6	F/30	ND	+	ST, Od, IT	Os	[51]	
16	c.3927_3931delAAAGA	p.Glu1309Aspfs*4	F/18	Gardner sy	-	-	Os		
17	c.4387_4390del	p.Arg1463fs	-/-	Gardner sy	ND	Dental anomalies	Os	[40]	
18	c.4292_4293delGA ^e^	p.Ser1465Trpfs*3	M/15	Gardner sy	ND	ST, Od, IT	-	[52]	
	c.4292_4293delGA ^e^	p.Ser1465Trpfs*3	M/66	Gardner sy	+	IT	-		
	c.4293_4294delAG	p.Ser1465Trpfs*3	F/31	Gardner sy	-	-	Os	[53]	
	c.4293_4294delAG ^f^	p.Ser1465Trpfs*3	F/28	Gardner sy	ND	-	Os		
	c.4293_4294delAG ^f^	p.Ser1465Trpfs*3	F/22	Gardner sy	ND	-	Os		
19	c.4510_4513del	p.Ser1505fs	-/-	Gardner sy	ND	-	Os, Dbi	[40]	
20	c.4609dup ^g^	p.Thr1537Asnfs*7	F/16	Gardner sy	ND	IT	Os	[16]	
	c.4609dup ^g^	p.Thr1537Asnfs*7	M/12	Gardner sy	ND	ST	Os		
21	c.4611_4612delAG	p.Glu1538Ilefs*5	-/-	Gardner sy	ND	ST	-	[40]	
22	c.4621C>T	p.Gln1541*	M/38	Gardner sy	-	IT, missing teeth	Os	[54]	
23	c.4652_4655delAAGA	p.Lys1551Argfs*13	-/-	Attenuated FAP	ND	ST	-	[45]	
24	c.4654_4655del	p.Glu1552Glyfs*6	-/-	Gardner sy	ND	Dental anomalies	Os	[55]	
25	c.4666del	p.Thr1556Leufs*9	M/25	Gardner sy	ND	ST, IT	Os, Dbi	[56]	
26	c.4668_4669insT	p.Ile1557*	-/-	Gardner sy	ND	-	Os	[40]	
27	c.4700C>G	p.Ser1567*	F/11	Gardner sy	ND	IT	-	[57]	
	c.4700C>G	p.Ser1567*	F/16	Gardner sy	ND	IT, Od	Os	[57]	
28	c.5722A>T	p.Asn1908Tyr	M/-	ND	ND	Mesiodens	-	[50]	
29	c.6127A>G	p.Ile2043Val	F/-	ND	ND	Mesiodens	-	[50]	
30	c.8383G>A ^f^	p.Ala2795Thr	M/-	ND	ND	Mesiodens	-	[50]	C-terminal region
	c.8383G>A ^f^	p.Ala2795Thr	M/-	ND	ND	Mesiodens	-	[50]	

a–g, represents family members; ST, supernumerary teeth; IT, impacted teeth; Os, osteoma; Od, odontoma; Dbi, dense bone island; Hs, hazy sclerosis; M, male; F, female; ND, not mentioned or no straightforward information; +, means the manifestation is present. -, means the manifestation is not present, except in “Gender/Age” column, where no information was available. Boldface refers to our cases.

**Table 2:** Frequency of dental and osseous manifestations in patients with FAP and in the general population.

Clinical Features	Frequency in Patients with FAP (%)	Frequency in the General Population (%)	Ref.
Osteomas	76.1	4.3	[62]
	62	14	[63]
	57.7	2.6	[58]
	46–93	4–16	[64]
	40	6.6	[65]
	60–80	1–2	[16]
Odontoma	26.9	0	[58]
	9.4–83.3	0–4	[64]
Supernumerary teeth	7.7	0	[58]
	11–27	0–4	[64]
Impacted teeth	11.5	3.8	[58]
	4–38	0–4	[64]
Dental anomalies	53.3	0	[65]

## Data Availability

The data presented in this study are available upon request from the corresponding author.
